# Pretreatment with AQP4 and NKCC1 Inhibitors Concurrently Attenuated Spinal Cord Edema and Tissue Damage after Spinal Cord Injury in Rats

**DOI:** 10.3389/fphys.2018.00006

**Published:** 2018-01-19

**Authors:** Xiaodong Yan, Juanfang Liu, Xiji Wang, Wenhao Li, Jingyuan Chen, Honghui Sun

**Affiliations:** ^1^Department of Orthopaedics, Tangdu Hospital, Fourth Military Medical University, Xi'an, China; ^2^Department of Clinical Aerospace Medicine, Fourth Military Medical University, Xi'an, China; ^3^Cadet Brigade, Fourth Military Medical University, Xi'an, China; ^4^Department of Occupational and Environmental Health, School of Public Health, Fourth Military Medical University, Xi'an, China

**Keywords:** spinal cord injury, spinal cord edema, AQP4, NKCC1, spinal cord tissue damage

## Abstract

Spinal cord injury (SCI) affects more than 2.5 million people worldwide. Spinal cord edema plays critical roles in the pathological progression of SCI. This study aimed to delineate the roles of aquaporin 4 (AQP4) and Na^+^-K^+^-Cl^−^ cotransporter 1 (NKCC1) in acute phase edema and tissue destruction after SCI and to explore whether inhibiting both AQP4 and NKCC1 could improve SCI-induced spinal edema and damage. Rat SCI model was established by modified Allen's method. Spinal cord water content, cerebrospinal fluid lactose dehydrogenase (LDH) activity, AQP4 and NKCC1 expression, and spinal cord pathology from 30 min to 7 days after SCI were monitored. Additionally, aforementioned parameters in rats treated with AQP4 and/or NKCC1 inhibitors were assessed 2 days after SCI. Spinal cord water content was significantly increased 1 h after SCI while AQP4 and NKCC1 expression and spinal fluid LDH activity elevated 6 h after SCI. Spinal cord edema and spinal cord destruction peaked around 24 h after SCI and maintained at high levels thereafter. Treating rats with AQP4 inhibitor TGN-020 and NKCC1 antagonist bumetanide significantly reduced spinal cord edema, tissue destruction, and AQP4 and NKCC1 expression after SCI in an additive manner. These results demonstrated the benefits of simultaneously inhibiting both AQP4 and NKCC1 after SCI.

## Introduction

Spinal cord injury (SCI) refers to damages to any part of the spinal cord, which causes changes in body functions below the injury site (Yu and He, 2015). The prevalence of traumatic SCI varies from 236 to 1,298 per million people globally (Furlan et al., 2013). Acute SCI is often a progressive process and the primary mechanical trauma is usually followed by a series of secondary injuries including ischemia, vascular changes, electrolyte disorders, edema, and loss of energy metabolism. Those changes in injured and adjacent areas after the acute post-injury phase can significantly increase the severity of SCI (Tator and Fehlings, 1991; Fan et al., 2013). Ischemia resulted from tissue compression, thrombosis, and vasospasm causes progressive neuronal death and aggravates other secondary injuries (Tator and Fehlings, 1991; Norenberg et al., 2004; Popa et al., 2010). Water was accumulated in spinal cord in the acute phase of SCI and associated with spinal edema formation and motor function recovery (Li and Tator, 1999; Sharma et al., 2005).

Lactate dehydrogenase (LDH) is a ubiquitously expressed soluble cytoplasmic enzyme which is released into extracellular space when the integrity of plasma membrane is compromised (Chan et al., 2013). The leakage of LDH into extracellular space has been widely used as a marker of cell death (Chan et al., 2013; Wang et al., 2014).

Aquaporin 4 (AQP4) was strongly expressed in rodent and human spinal cord tissues (Nesic et al., 2006; Misu et al., 2007; Shibuya et al., 2008), which allowed fast water movement in and out of neurons and astrocytes (Yang et al., 2008). Spinal cord AQP4 level was elevated after chronic SCI and its level was correlated with spinal cord water content (Nesic et al., 2006). Overexpressing AQP4 in glia promoted brain edema after acute water intoxication (Yang et al., 2008) but the studies with AQP-null mice yielded conflicting conclusions on whether AQP4 protected against or exacerbated SCI induced spinal edema and neuronal dysfunction (Saadoun et al., 2008; Yang et al., 2008; Kimura et al., 2010; Wu et al., 2014b). Nevertheless, it has been established that AQP4 played a critical role in the pathological processes after spinal cord injuries.

Cation chloride cotransporters, Na^+^-K^+^-Cl^−^ cotransporter 1 (NKCC1), playing significant roles in cellular ionic homeostasis and the accumulation of intracellular water (Lu et al., 2008), was transiently overexpressed in spinal cord tissues after SCI (Hasbargen et al., 2010). In brain, NKCC1 mediated cerebral edema and neuron death after traumatic brain injury (Lu et al., 2008; Hui et al., 2016). Kinase Wnk1 (With no lysine) regulated the spinal cord edema and chronic phase neuropathic pain after SCI via modulating the expression and phosphorylation of NKCC1 (Ahmed et al., 2014). Pretreatment with AQP4 inhibitor TGN-020 significantly reduced focal cerebral ischemia induced brain edema (Igarashi et al., 2011) whereas bumetanide was shown to attenuate brain edema after traumatic brain injury (Hui et al., 2016). Based on these observations, we hypothesized that it could be beneficial to simultaneously inhibiting AQP4 and NKCC1 after SCI. This study aimed to investigate whether concurrently blocking NKCC1 and AQP4 could additively or synergistically inhibit spinal cord edema and tissue damage after SCI.

## Materials and methods

### Establishment of acute SCI rat model

All procedures were approved by the Institute of Animal Care and Use Committee of Tangdu Hospital, Fourth Military Medical University. All methods were performed in accordance with “the Regulations on the Care and Usage of Laboratory Animals” issued by the Ministry of Science and Technology of the People's Republic of China. Total 100 adult female (8-week old) Sprague-Dawley (SD) rats were purchased from Laboratory Animal Center of Fourth Military Medical University (Xi'an, China). They were housed in facility maintained at 22–24°C, 45–55% humidity, 12:12 h light: dark cycle with free access to food and water. All rats were allowed to acclimate for 1 week before experiments.

A rat SCI model was established with a modified Allen's method (Chen et al., 2013). Briefly, rats were anesthetized by intraperitoneal injection of pentobarbital sodium at 50 mg/kg and fixed on a stereotaxic apparatus (DL Naturegene, Beijing, China). After shearing hair and disinfecting with 75% ethanol, an about 3 cm incision was made at the 12th rib, skin was opened and muscles around the spinous process were separated. The spinal cord was exposed by removing the T11-T12 vertebral plate and opening the canalis spinalis to expose dura mater spinalis. The T10 and T13 spinous processes were clamped to ensure the location of injury. The contusion was introduced with a customized IH-0400 Spinal Cord Impactor (PSI, Lexington, KY, USA) at the spinal cord corresponding to the T12 spinous process. The rod was 20 g and 2.5 mm in diameter. The striking force was 20 × 2.5 g•cm and contact time was 1 s. Then the muscles were realigned and wound was closed. The rats in sham group were undergone the same procedure except spinal cord contusion. The body temperature was maintained at 36.0–37.0°C with incandescent lamp and rectal thermometer monitoring throughout the operation. After operation, rats were individually housed and given tetracycline (10 mg/kg, SQ) and buprenorphine (0.05 mg/kg, SQ) twice daily for 3 days. The bladder was emptied manually three times daily until rats were capable for reflex bladder emptying. Nine rats were excluded from further experiments due to death (2 rats from time-course study, 1 from bumetanide group), insufficient injury (1 rat from SCI group, locomotor function recovered in <24 h), and no or too little eating/drinking (4 rats from time-course study, 1 from TGN-020 + bumetanide group). Rats were euthanized at specified time from 0 to 7 days (*n* = 7 at each time point) by intraperitoneally administering 150 mg/kg pentobarbital with 25 mg/kg phenytoin. Spinal fluid was collected by direct cistern magna puncture (Mahat et al., 2012). Spinal cord around the striking site (2 cm in length) was collected and divided into 4 portions across the epicenter for assessing water content (rostral ventral section), for RNA work (rostral dorsal section), for protein work (caudal ventral section), and fixed for histological works (caudal dorsal section).

For drug treatment, 200 mg/kg of TGN-020 (Sigma-Aldrich, St. Louis, MO) (Igarashi et al., 2011) and/or 0.3 mg/kg of Bumetanide (Sigma-Aldrich) (Cleary et al., 2013), dissolved in 0.1 ml normal saline was administered intra-peritoneally 15 min before induction of SCI (*n* = 7 in each treatment group). Those animals were sacrificed 48 h after SCI.

### BBB locomotor rating scale

The 21-point (0–21) Basso, Beattie, and Bresnahan (BBB) locomotor rating scale was used to assess the behaviors of rats before and 0.5, 1, 3, and 7 days after injury (*n* = 7), which was based on the observation of hindlimb movements of a rat freely moving in an open field (Basso et al., 1995, 1996). During the evaluation, rats were allowed to freely walk on the open field for 4 min.

### Determination of spinal cord edema

The level of spinal cord edema was expressed by water content in the spinal cord tissue. A 1.5-cm spinal cord tissue band centered around the injury site was weighed for the wet weight and weighed again for the dry weight after it was dried for 24 h in an 80°C oven. The water content in the spinal cord tissue was calculated as (wet weight–dry weight)/wet weight × 100% (Li et al., 2016; Cabrera-Aldana et al., 2017).

### LDH activity assessment

LDH activity in spinal fluid was analyzed using an LDH activity assay kit (NJJCBio, Nanjing, China) according to manufacturer's instruction. Briefly, 20 μl of spinal fluid was mixed with 250 μl matrix buffer, 50 μl coenzyme I working solution and incubated at 37°C for 15 min. Then 250 μl 2,4-dinitrophenylhydrazine was mixed in and the mixture was incubated another 15 min at 37°C. The reaction was stopped by 250 μl of 0.4 mol/L NaOH and left at room temperature for 3 min before read at 440 nm. Spinal fluid LDH activity (U/ml) = (OD_Sample_−OD_Control_)/(OD_Standard_−OD_Blank_) ^*^ Standard Concentration ^*^ dilution factor/sample volume (ml).

### Reverse transcription PCR and quantitative real-time PCR (qPCR)

Total RNA from rat spinal cord tissues was extracted with RNeasy Mini Kit (Qiagen, Shanghai, China) according to manufacturer's manual. The first strand cDNA was synthesized with a reverse transcription kit from Tiangen Biotech (Beijing, China) according to manufacturer's protocol. Quantitative real-time PCR was performed with TransStart Top Green qPCR SuperMix from TransGen (AQ131-01, Beijing, China) on a ABI 7300 (Applied Biosystems, Foster City, CA) with primers CTCAACGCACCTAACAGGGA and GACGGAAGGCGGTTTTCAAG for NKCC1; CTGGGGGCAGGCAATGAGAG and GGGAGGTCCACACTTACCCC for AQP4; and GATGTGGATCAGCAAGCAGGA and AAAACGCAGCTCAGTAACAGTCC for Actb. The reaction program was consisted of 95°C for 3 min followed by 40 cycles of 95°C 30 s, 55°C 20 s, and 72°C 20 s. The relative mRNA levels were calculated by 2^−ΔΔCt^ method with Actb as the internal control.

### Western blot

The total protein of the spinal cord tissues was extracted using RIPA lysis buffer. Total proteins of each group (40 μg) were resolved in 8% sodium dodecyl sulfate polyacrylamide gel electrophoresis (SDS-PAGE) and then transferred onto polyvinylidenefluoride (PVDF) membranes (Millipore, Bedford, MA). After the membranes were blocked in5% non-fat milk at room temperature for 30 min, they were incubated with anti-AQP4 (ab46182, Abcam, Cambridge, MA), anti-NKCC1 (ab59791, Abcam), or anti-β-actin (A2066, Sigma, St Louis, MO) antibody at 4°C overnight. After 3 washes with PBST, the membranes were incubated with proper horseradish peroxidase-conjugated goat anti-rabbit IgG antibody at room temperature for 60 min, and visualized with the enhanced chemiluminescence (ECL) substrate (ThermoFisher, Shanghai, China). The images were scanned and analyzed with ImageJ (NIH, Bethesda, MD).

### Histological examinations

The histology of spinal cord was evaluated by hematoxylin and eosin (HE) staining and Pischinger methylene blue staining (Khedkar et al., 2012). The left ventral section of spinal cord was fixed in formalin and embedded into paraffin. The 6 μm coronal sections were cut from the tissue block and stained with HE stain or Pischinger's methylene blue before evaluated microscopically with an Olympus IX71 Inverted Fluorescence Phase Contrast Microscope (Olympus, Shanghai, China). The areas of cavities were assessed using Digimizer software (MedCalc Software bvba, Ostend, Belgian) and number of dendrites was quantified with ImageJ software (NIH, Bethesda, MD).

### Immunohistochemical staining

Paraffin-embedded spinal cord tissue at injury site was cut at a thickness of 6 micron. Slides were deparaffinized and rehydrated by washing in xylene and passing through ethanol gradient. Endogenous peroxidase activity was quenched with freshly made 0.3% hydrogen peroxide and antigens were heat retrieved in citrate buffer. Slides were blocked with normal goat serum at 37°C for 30 min, incubated with antibody against AQP4 (ab46182, Abcam) or NKCC1 (ab59791, Abcam) overnight at 4°C, rinsed with 0.01 M PBS 3 min for 3 times, incubated with FITC- (for AQP4) or Cy5- (for NKCC1) conjugated goat anti-rabbit IgG antibodies at 37°C for 30 min, conterstained with DAPI. The slides were dehydrated with alcohol gradient, xylene cleared, and mounted with neutral gum. The images were obtained with an Olympus IX71 Microscope.

### Statistical analysis

The data were expressed as mean ± standard error (SE). The sample size was 7 rats in each group. Statistical analyses were performed using the SPSS16.0 (IBM, Chicago, IL). The differences between groups were analyzed using student *t*-tests or one-way analysis of variance (ANOVA) followed by post-hoc bonferroni test. *P*-values less than 0.05 were considered statistically significant.

## Results

### Acute SCI caused spinal cord edema and neuronal loss

**Figure 1:**
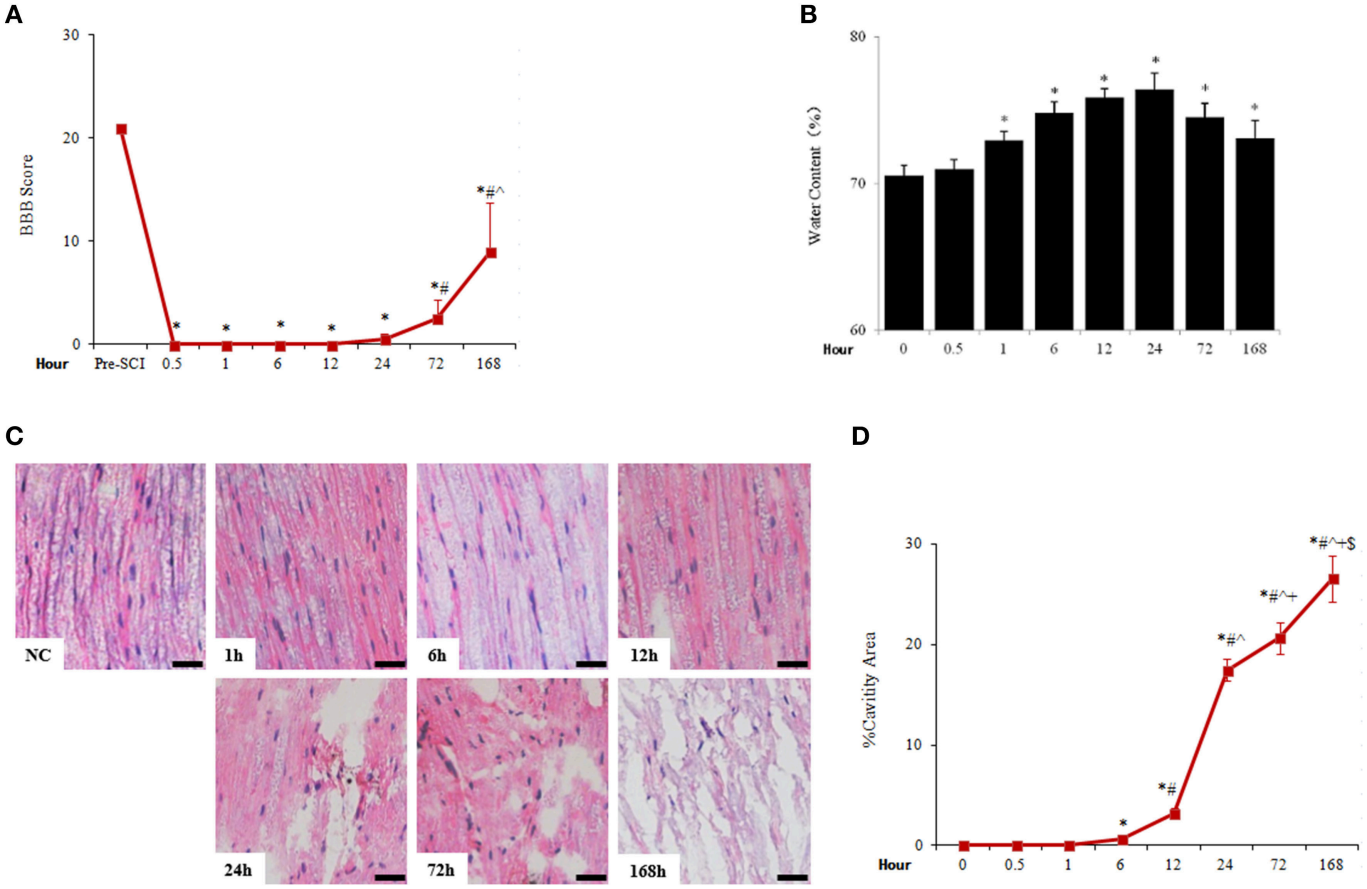
Acute SCI caused rapid and sustained increase of spinal cord water content and tissue damage. **(A)** The change of BBB score after SCI. *N* = 7. ^*^p < 0.05 compared to pre-SCI, ^#^*p* < 0.05 compared to 0.5, 1, 6, 12, and 24 h, ˆ*p* < 0.05 compared to 72 h. **(B)** Spinal cord water content was assessed by the percentage of the difference of wet weight and dry weight. *N* = 7. ^*^p < 0.05 compared to control (0 h). **(C)** Pathological changes of spinal cord adjacent to injury sites were examined by HE staining. Apparent cavities were seen 6 h after spinal cord injuries (scale bar 50 μm). NC, sham. **(D)** Quantitative analysis of cavity spaces. *N* = 7. ^*^p < 0.05 compared to control (0 h), ^#^*p* < 0.05 compared to 6 h, ˆ*p* < 0.05 compared to 12 h, ^+^*p* < 0.05 compared to 24 h, ^$^*p* < 0.05 compared to 72 h.

The locomotor activity of rats was lost after immediately SCI shown by the plunge of BBB score to near 0 and remaining there for about a day before visible joint movements. BBB score showed modest increase from day 1 to day 7 after SCI (Figure 1A).

Traumatic SCI caused rapid occurrence of spinal edema which peaked at 24 h and lasted at least 7 days after SCI. The spinal cord water content was seen significantly increased 1 h (72.92 ± 0.59%), peaked at 24 h after SCI (76.41 ± 1.09%), and reduced thereafter but kept at significantly higher levels than that of uninjured spinal cord (70.54 ± 0.71%; Figure 1B). Accompanied by spinal cord edema, the histology of spinal cord tissue was gradually distorted and damaged as cavitations appeared 6 h after injury, then the destruction of spinal cord structure became progressively profound during the duration monitored (7 days; Figures 1C,D).

**Figure 2:**
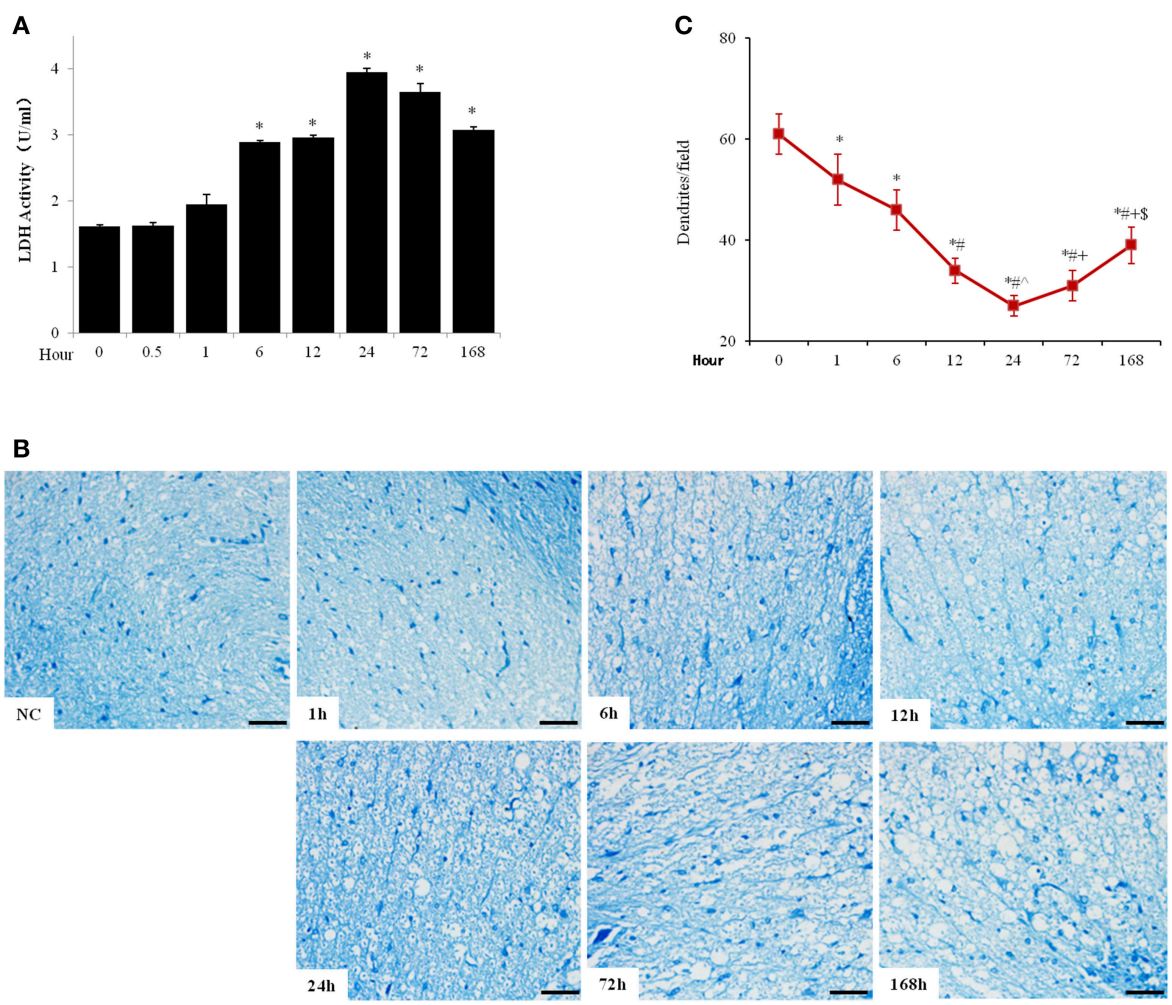
Acute SCI elicited cell death and tissue destruction in spinal cord. **(A)** Spinal fluid LDH activity was analyzed with commercial LDH activity assay kit. **(B)** Pischinger's methylene blue staining showed decrease of dendrites and appearance of cavities 6 h after SCI and further progressed until at least 7 days after injury (scale bar 50 μm). NC, sham. **(C)** Quantitative analysis of number of dendrite per field (200x). *N* = 7. ^*^p < 0.05 compared to control (0 h), ^#^*p* < 0.05 compared to 6 h, ˆ*p* < 0.05 compared to 12 h, ^+^*p* < 0.05 compared to 24 h, ^$^*p* < 0.05 compared to 72 h.

Meanwhile, the LDH activity in cerebrospinal fluid was significantly increased from 1614.67 ± 26.08 to 2892.33 ± 25.97 U/g protein 6 h after SCI and peaked at 24 h after injury (3953.46 ± 58.50 U/g protein), which then reduced but maintained at high levels until at least 7 days after SCI (Figure 2A). Pischinger's methylene blue staining showed reduced number of dendrites and increase of tissue cavities from 6 h after SCI and deteriorated until at least 7 days after SCI (Figures 2B,C).

### SCI induced the upregulation of AQP4 and NKCC1 expression in spinal cord

**Figure 3:**
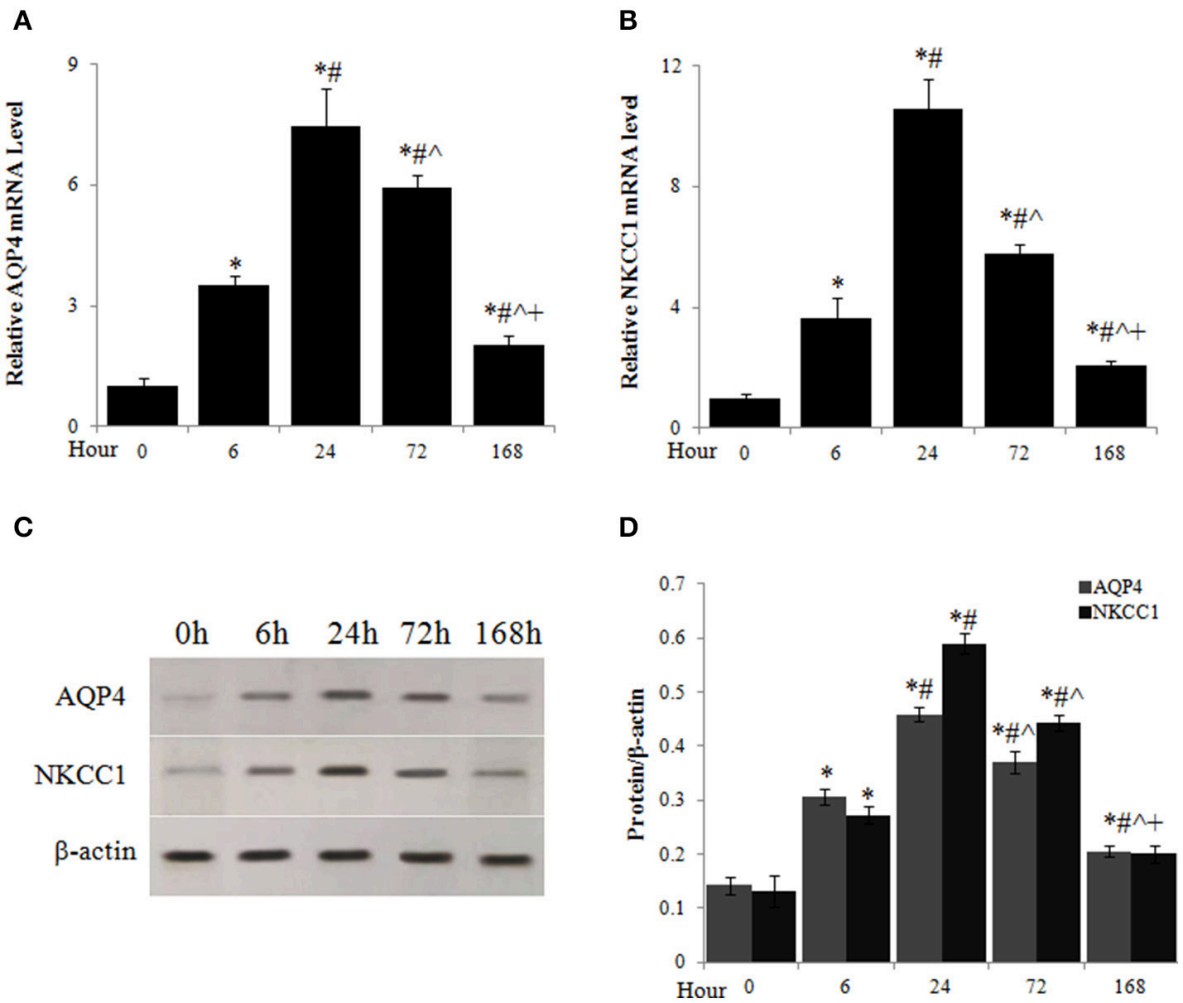
Acute SCI caused upregulation of AQP4 and NKCC1 expression. The mRNA levels of AQP4 **(A)** and NKCC1 **(B)** at different time points after SCI were analyzed by quantitative PCR. **(C)** AQP4 and NKCC1 protein levels at different time points after SCI were assessed by western blot. **(D)** Quantitative analysis of the changes of AQP4 and NKCC1 protein in spinal cord after SCI. *N* = 7. ^*^p < 0.05 compared to control (0 h), ^#^*p* < 0.05 compared to 6 h, ˆ*p* < 0.05 compared to 24 h, ^+^*p* < 0.05 compared to 72 h.

The mRNA levels of AQP4 (Figure 3A) and NKCC1 (Figure 3B) of spinal cord were significantly elevated 6 h, peaked 24 h, and decreased from the peak but maintained at high levels until 7 days after injury. Similarly, AQP4 and NKCC1 protein rapidly accumulated in spinal cord after SCI, peaked around 24 h after SCI, and reduced thereafter but maintained at significantly higher levels compared to uninjured spinal cords (0 h) through at least 7 days after injury (Figures 3C,D).

### Blocking AQP4 and NKCC1 additively reduced SCI-induced spinal cord edema and damage

**Figure 4:**
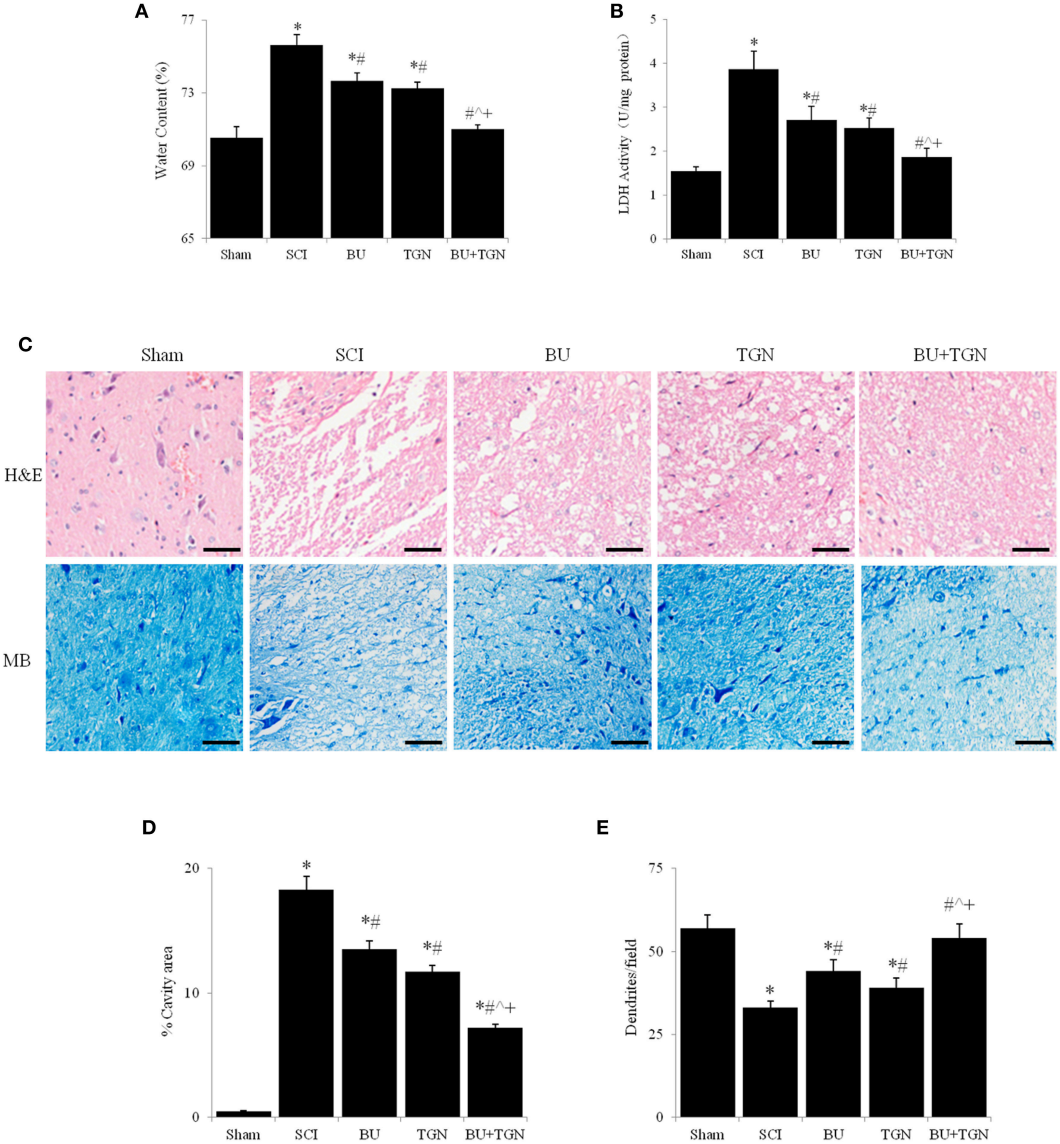
Blocking AQP4 and / or NKCC1 reduced spinal cord water content and tissue destruction 48 h after SCI. Spinal cord water content **(A)** and LDH activity of cerebrospinal fluid **(B)** were significantly reduced by TGN-20 and bumetanide after SCI, TGN-20, and bumetanide additively reduced spinal cord edema and cerebrospinal LDH activity. **(C)** HE and methylene blue stainings showed that TGN-20 and bumetanide relieved SCI-induced tissue degeneration (scale bar 50 μm). Quantitative analyses of cavity area **(D)** and dendrite numbers per field (200x) **(E)** NC, normal control (uninjured spinal cords); BU, bumetanide; TGN, TGN-020; HE, hematoxylin and eosin staining; MB, methylene blue staining. *N* = 7. ^*^p < 0.05 compared to NC; ^#^*p* < 0.05 compared to SCI; ˆ*p* < 0.05 compared to BU; ^+^*p* < 0.05 compared to TGN.

Rats receiving AQP4 inhibitor TGN-020 or NKCC1 blocker bumetanide before SCI had significantly lower spinal cord water content compared to untreated SCI rats, and concomitant administration of TGN-020 and bumetanide resulted in further reduction of spinal cord edema (Figure 4A). Meantime, TGN-20 and or bumetanide significantly inhibited SCI-caused increase of cerebrospinal fluid LDH activity from about 30% to more than 50% (Figure 4B). Consequently, SCI caused spinal cord tissue destruction was substantially reduced by TGN-020, or bumetanide, or both (Figures 4C–E). TGN-020 and bumetanide reduced cavity area by about 30% respectively, and the combination of TGN-020 and bumetanide reduced the cavity area by more than 50% (Figures 4C,D). Meanwhile, TGN-020 and bumetanide significantly reduced SCI-caused loss of dentrites in monotherapy or combinatorial treatment (Figures 4C,E).

### AQP4 and NKCC1 functionally interacted with each other after acute SCI

**Figure 5:**
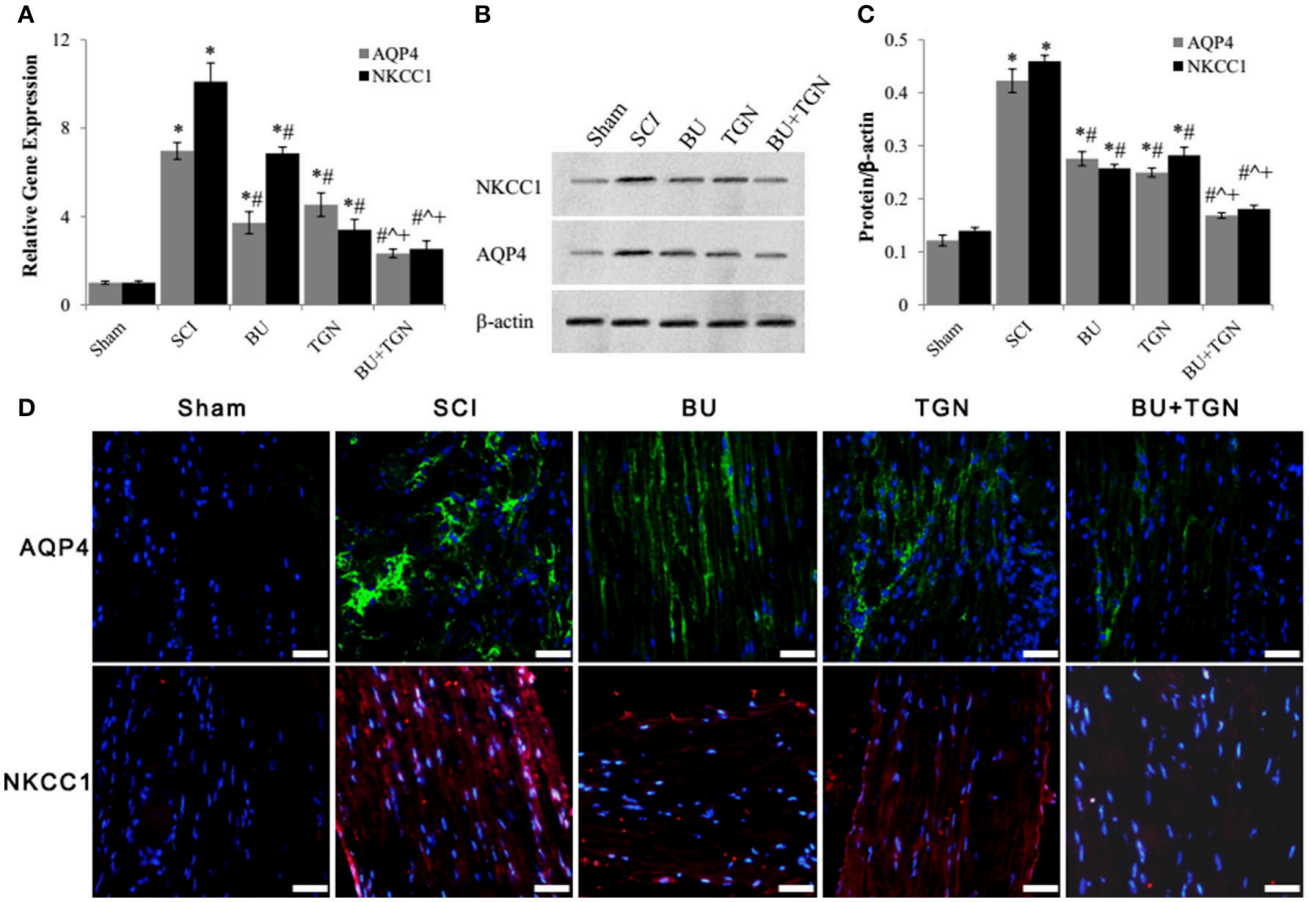
Bumetanide and TGN-020 inhibited SCI-induced upregulation of AQP4 and NKCC1. **(A)** The mRNA levels of AQP4 and NKCC1 of rat spinal cord 48 h after SCI were analyzed by qPCR. **(B)** The changes of AQP4 and NKCC1 protein levels in rat spinal cord 48 h after SCI with or without TGN-020 and/or bumetanide treatment were assessed by western blot. **(C)** Quantitative analysis of AQP4 and NKCC1 protein levels in SCI rats after TGN-020 and/or bumetanide treatments. **(D)** Histoimmunofluorescence detection of changes of AQP4 (green) and NKCC (red) protein levels of injured rat spinal cord tissue treated with TGN-020 and/ or bumetanide (scale bar 50 μm). NC, normal control (uninjured spinal cords); BU, bumetanide; TGN, TGN-020. *N* = 7. ^*^p < 0.05 compared to NC; ^#^*p* < 0.05 compared to SCI; ˆ*p* < 0.05 compared to BU; ^+^*p* < 0.05 compared to TGN.

Blockade of AQP4 with TGN-020 not only inhibited SCI induced upregulation of AQP4 mRNA (Figure 5A) and protein (Figures 5B–D) levels of spinal cord, but reduced NKCC1 expression as well (Figure 5). Similarly, bumetanide inhibited both SCI-induced NKCC1 and AQP4 overexpression (Figure 5). Blocking both AQP4 and NKCC1 resulted in markedly decrease of their mRNA and protein levels after SCI compared to treatment with either bumetanide or TGN-020 alone (Figures 5A–D).

## Discussion

The current data showed that water channel AQP4 and influent Na^+^-K^+^-Cl^−^ cotranporter NKCC1 were upregulated in spinal cord after acute SCI, which in turn cooperatively contributed to spinal cord edema, spinal cord neuron death, and destruction of spinal cord tissues. Blocking either AQP4 or NKCC1 with their specific inhibitor partially relieved the damaging effects of SCI in rats but blocking AQP4 and NKCC1 with TGN-020 and bumetanide concurrently produced significantly stronger protection against SCI-induced edema and tissue destruction of spinal cord than either one of them.

The accumulation of blood and/or water (edema) in spinal cord after SCI raises intrathecal pressure, which may result in greater tissue damage and exacerbate locomotor function loss (Leonard et al., 2015). Controlling or resolving edema has been extensively studied as an effective therapy for SCI (Zu et al., 2014; Hu et al., 2015; Zhang et al., 2015). Either chemicals (Zu et al., 2014; Zhang et al., 2015) or surgical intervention (Hu et al., 2015) resulted reduction of spinal cord edema after SCI was mediated at least partially by AQP4.

AQP4 expression was elevated after SCI and mediated spinal cord edema which was inhibited by treatments inhibiting AQP4 expression or AQP4 activity (Wu et al., 2014a; Huang et al., 2015; Hu et al., 2015). Pretreatment with TGN-020 resulted in more than 40% reduction in brain edema and more than 30% reduction in the size of cortical infarction in a mouse model of focal cerebral ischemia (Igarashi et al., 2011). It also has been shown that upregulation of AQP4 mediated cerebral edema and neuronal death after traumatic brain injury (Kapoor et al., 2013; Xiao and Hu, 2014; Zhang et al., 2016) and inhibiting AQP4 expression with safranal led to attenuation of edema, reduced apoptosis, and inhibition of inflammation after traumatic SCI (Zhang et al., 2015). Surprisingly, partial sciatic nerve transaction and chronic constriction injury on the left sciatic nerve induced AQP4 upregulation and astrocyte swelling and activation in spinal cord (Oklinski et al., 2015). Based on the functional interaction between AQP4 and NKCC1 in the central nerve system, it was shown beneficial to simultaneously inhibit injury-induced upregulation of AQP4 and NKCC1 after traumatic brain injury (Zhang et al., 2016). The current study was a proof of concept that concurrently blocking AQP4 and NKCC1 after SCI could have better effect than inhibiting either one of them. Further studies were warranted to elucidate the mechanism, effects on motor function recovery, proper regimen, possible adverse effects, and appropriate agents for the combination of AQP4 and NKCC1 antagonists to treat spinal cord injuries.

NKCC1 has been shown to be upregulated (Cramer et al., 2008; Côté et al., 2014) and activated by WNK1 (with no lysine 1) (Ahmed et al., 2014) after SCI, which mediated astrocyte swelling (Jayakumar et al., 2011) and neuropathic pain (Cramer et al., 2008; Ahmed et al., 2014). NKCC1 specific antagonist bumetanide was able to alleviate neuropathic pain (increased withdrawal latency time) after SCI (Cramer et al., 2008; Ahmed et al., 2014) and inhibit brain edema and neuronal death after traumatic brain injuries (Lu et al., 2008; Hui et al., 2016). Inhibiting NKCC1 activity with bumetanide or silencing NKCC1 expression significantly suppressed traumatic brain injury induced intracellular Na^+^ increase, neuronal apoptosis, brain edema, and improved neurological function (Hui et al., 2016). NKCC1 antagonist bumetanide significantly reduced AQP4 protein level after traumatic brain injury (Zhang et al., 2016). The current data demonstrated that not only inhibition of NKCC1 downregulated SCI-induced AQP4 expression but vice versa also. Inhibiting NKCC1 and AQP4 activities with bumetinade and TGN-020 cooperatively reduced both spinal cord edema and spinal cord tissue damage resulted from SCI-induced neuronal death and intracellular swelling. Moreover, NKCC1 and AQP4 had similar expression pattern after acute SCI, which was consistent with previous report that NKCC1 was identified as one of the gene positively correlated with AQP4 in SCI rats (Nesic et al., 2005).

In conclusion, this study demonstrated that AQP4 and NKCC1 shared similar expression patterns after acute SCI. AQP4 and NKCC1 functionally interacted with each other and influenced the expression of each other, which mediated acute SCI induced disruption of ion and water homeostasis, cytotoxic edema, and neuronal death. Simultaneous inhibition of AQP4 and NKCC1 provided better protection against spinal cord edema and spinal cord tissue destruction after acute SCI.

## Author contributions

XY: Established SCI model, performed histology study, analyzed data; JL: Performed qPCR and western blot; XW: Performed immunofluorescence; WL: Performed ELISA; JC and HS: Conceived the project, obtained grants, wrote the manuscript; All authors reviewed and agreed the manuscript.

### Conflict of interest statement

The authors declare that the research was conducted in the absence of any commercial or financial relationships that could be construed as a potential conflict of interest. The reviewer JG and handling Editor declared their shared affiliation.
